# Whole-Genome Sequencing of a Canine Family Trio Reveals a *FAM83G* Variant Associated with Hereditary Footpad Hyperkeratosis

**DOI:** 10.1534/g3.115.025643

**Published:** 2016-01-08

**Authors:** Shumaila Sayyab, Agnese Viluma, Kerstin Bergvall, Emma Brunberg, Vidhya Jagannathan, Tosso Leeb, Göran Andersson, Tomas F. Bergström

**Affiliations:** * Department of Animal Breeding and Genetics, Swedish University of Agricultural Sciences, 750 07 Uppsala, Sweden; † Research Center for Modeling and Simulation, National University of Sciences and Technology, Sector H-12, Islamabad, Pakistan; ‡ Department of Clinical Sciences, Swedish University of Agricultural Sciences, 750 07 Uppsala, Sweden; § Norwegian Centre for Organic Agriculture, NO-6630 Tingvoll, Norway; ** Institute of Genetics, University of Bern, 3001 Bern, Switzerland

**Keywords:** FAM83G, NGS, WGS, canine, whole-genome-sequencing

## Abstract

Over 250 Mendelian traits and disorders, caused by rare alleles have been mapped in the canine genome. Although each disease is rare in the dog as a species, they are collectively common and have major impact on canine health. With SNP-based genotyping arrays, genome-wide association studies (GWAS) have proven to be a powerful method to map the genomic region of interest when 10–20 cases and 10–20 controls are available. However, to identify the genetic variant in associated regions, fine-mapping and targeted resequencing is required. Here we present a new approach using whole-genome sequencing (WGS) of a family trio without prior GWAS. As a proof-of-concept, we chose an autosomal recessive disease known as hereditary footpad hyperkeratosis (HFH) in Kromfohrländer dogs. To our knowledge, this is the first time this family trio WGS-approach has been used successfully to identify a genetic variant that perfectly segregates with a canine disorder. The sequencing of three Kromfohrländer dogs from a family trio (an affected offspring and both its healthy parents) resulted in an average genome coverage of 9.2X per individual. After applying stringent filtering criteria for candidate causative coding variants, 527 single nucleotide variants (SNVs) and 15 indels were found to be homozygous in the affected offspring and heterozygous in the parents. Using the computer software packages ANNOVAR and SIFT to functionally annotate coding sequence differences, and to predict their functional effect, resulted in seven candidate variants located in six different genes. Of these, only *FAM83G:*c155G > C (p.R52P) was found to be concordant in eight additional cases, and 16 healthy Kromfohrländer dogs.

To identify genetic changes responsible for inherited diseases, the domestic dog is an attractive model. A successful approach to identify mutations associated with disease has been to perform case-control studies based on genome-wide association studies (GWAS) using large-scale SNP-array genotyping, followed by statistical association analysis. For autosomal recessive diseases caused by a single gene mutation, a sample collection of 10–20 affected dogs, and an equal number of healthy dogs is often needed. To recruit sufficient samples for rare diseases is difficult and time-consuming, and the associated region is typically very large, containing 0.5–1 million bp that needs to be resequenced for identification of disease-associated genetic variants. Here we used a different approach based on whole-genome sequencing of three dogs—a family trio where the offspring was diagnosed with Hereditary Footpad Hyperkeratosis (HFH) and the two healthy parents. HFH is a monogenic disease, and we presumed that both parents were heterozygous and that the affected offspring was homozygous for the associated mutation. A mutation in the affected individual was detected that results in a change of an amino acid in the *FAM83G* gene that was predicted to negatively influence the function of the encoded protein. Perfect genetic concordance between homozygosity for this mutation and HFH was confirmed in a larger set of dogs affected by the disease.

In recent years, whole-genome sequencing (WGS) and whole-exome sequencing (WES) of family trios has emerged as a powerful approach to identify mutations associated with inherited human diseases. This is the result of a rapid technological development of next generation sequencing (NGS) methods, and subsequently a significant reduction in cost for sequencing an individual’s genome. Both WGS and WES, generally refers to resequencing of an individual genome or exome, and the reads are aligned to an appropriate reference genome sequence rather than being assembled *de novo*. An attractive aspect of NGS is the unbiased and large-scale detection of genetic variation, including single base pair substitutions, insertion/deletions and large structural variation resulting in inherited diseases and disorders. The majority of human studies have relied on WES and different sequence capture and enrichment techniques. In the first proof-of-concept study, WES was used to identify *MYH3* as the disease-causing gene in four unrelated individuals affected by a rare dominantly inherited disorder, the Freeman-Sheldon Syndrome (Ng *et al.* 2009). A large number of genes for human Mendelian diseases have now been identified using WES (for review see: Bamshad *et al.* 2011; Boycott *et al.* 2013; Chong *et al.* 2015; Shen *et al.* 2015). In contrast, WES methodologies have not been used extensively in canine Mendelian disease genetic research.

Since the publication of the canine reference genome in 2005 (Lindblad-Toh *et al.* 2005), the most common approach for identifying canine genetic risk factors has been to use SNP-based genotyping and genome-wide association studies (GWAS). The first proof-of-principle study used a two-tiered GWAS approach with two breeds where the haplotype associated with white coat color was identified (Karlsson *et al.* 2007). Since then, case-control studies based on SNP genotyping and GWAS in a single breed has been used extensively, and frequently resulted in the successful identification of genes associated with traits and diseases. For Mendelian traits, these studies have typically involved SNP-based genotyping of 10–20 unrelated cases and an equal number of healthy controls. With the extensive degree of linkage disequilibrium (LD) generally observed within dog breeds, these studies often result in an associated region spanning between 0.5 and 1 Mb that requires fine-mapping with additional SNPs and targeted resequencing to identify a candidate mutation (for review, see Karlsson *et al.* 2007; Karlsson and Lindblad-Toh 2008; Parker *et al.* 2010). The recent update of the canine reference genome (CanFam3.1) and the development of more dense SNP-arrays such as the 170K CanineHD BeadChip (Illumina), have further increased the efficacy of this experimental design (Hoeppner *et al.* 2014; Vaysse *et al.* 2011). It is, however, not unusual that the associated region spans several megabases (*e.g.*, Downs *et al.* 2014; Vernau *et al.* 2013).

In recent years, several canine disease-associated mutations have been identified with a combination of GWAS and WGS (Frischknecht *et al.* 2013; Gerber *et al.* 2015; Jagannathan *et al.* 2013; Kyostila *et al.* 2015; Owczarek-Lipska *et al.* 2013; Wolf *et al.* 2015). With this approach, GWAS performed on only a handful of individuals is used for defining an associated region in the genome followed by WGS of one affected dog. The critical interval of the affected dog’s genome sequence is then compared to nonaffected dogs to find candidate mutations for the disease. In rare diseases, where only a few affected and unaffected individuals are available from a small family pedigree, successful identification of candidate mutations has also been achieved by a combination of SNP-based linkage analysis and WGS (Kyostila *et al.* 2015; Steffen *et al.* 2015; Willet *et al.* 2015). The power of WGS in candidate gene studies is further illustrated by two studies where the genomes of single individuals affected by a neuronal ceroid lipofuscinosis were sequenced, and the data mined for candidate mutations (Gilliam *et al.* 2015; Guo *et al.* 2014). This led to the successful identification of a frame shift mutation in the *CLN5* gene in Golden retriever (Gilliam *et al.* 2015), and a nonsense mutation in the *CLN8* gene in a mixed breed dog with Australian shepherd ancestry (Guo *et al.* 2014).

In a situation where no obvious candidate genes are known, an alternative approach is to use family-based WGS for gene discovery of rare variants associated with Mendelian diseases and traits. In comparison to WES, analysis of the complete genome eliminates biases associated with capture-based enrichment technologies and allows the detection of noncoding variants that may be associated with a trait. In addition, the WGS data will remain useful even when the annotation of the canine reference genome is further improved. The Ion Proton System (Thermo Fisher Scientific/Life Technologies) has previously been evaluated for WGS of dogs (Viluma *et al.* 2015), and in the present study we applied the technology to perform WGS of a family trio as a straightforward approach to discover genetic variants associated with an autosomal recessive disease termed Hereditary Footpad Hyperkeratosis (HFH) segregating in the Kromfohrländer breed. This allowed the identification of a missense variant in *FAM83G* (c155G > C, p.R52P) associated with HFH.

During the progress of our study, Drögemüller and colleagues independently identified the same missense variant using a combination of a two-tiered GWAS in two breeds (42 Kromfohrländer dogs, 13 cases and 29 controls, and 31 Irish Terriers, 10 cases and 21 controls), and resequencing of a single affected Kromfohrländer individual (Drögemüller *et al.* 2014). Combined, the use of different canine cohorts and experimental designs strengthens the conclusion that the genetic variant in *FAM83G* is associated with HFH. It further demonstrates the efficacy of the trio-based WGS approach, and serves as a proof-of-concept for detecting genetic variants associated with development of canine monogenic diseases.

## Materials and Methods

### Canine subjects

Kromfohrländer dogs were recruited via the breed association and breeders of Kromfohrländer dogs. A boarded veterinary dermatologist clinically examined the dogs. Pedigrees from all dogs were collected. Dogs with a history of hyperkeratosis affecting all four paw pads since juvenility, and with clinical lesions compatible with the disease, with hyperkeratotic, firm and cracking pads were diagnosed as cases, whereas dogs with clinically normal paw pads were included as healthy controls. Biopsies, when taken from cases, revealed histopathological changes typical for paw pad hyperkeratosis. All samples were obtained with informed dog owner consent. Ethical approval was granted by the Swedish Animal Ethical Committee Dnr C12/15.

Whole blood was collected from nine cases and 18 healthy Kromfohrländer dogs into EDTA tubes. Genomic DNA was extracted from peripheral blood leukocytes, using 1 ml blood on a QIAsymphony SP instrument using the QIAsymphony DSP DNA Kit (Qiagen, Hilden, Germany).

### Ion proton sequencing

A 1-µg sample of gDNA was fragmented using the Covaris S2 instrument (Covaris Inc.), and library preparation was performed using the Ion Xpress Plus Fragment Library Kit for AB Library Builder System (Thermo Fisher Scientific/Life Technologies) followed by five cycles of amplification. Emulsion PCR was done on the Ion OneTouch 2 system with Ion PI Template OT2 200 Kit v2 chemistry (Thermo Fisher Scientific/Life Technologies). Enrichment was conducted using the Ion OneTouch ES (Thermo Fisher Scientific/Life Technologies). Samples were loaded on two Ion PI chips Kit v2, and sequenced on the Ion Proton System using Ion PI Sequencing 200 Kit v2 chemistry (200 bp read length, Thermo Fisher Scientific/Life Technologies).

### Next-generation sequence analysis

Reads were aligned to the canine reference sequence assembly CanFam3.1 (Hoeppner *et al.* 2014) using TMAP, an implementation of BWA, SSAHA, and Supermaximal Exact Matching (Li and Durbin 2010, 2009; Ning *et al.* 2001; Li 2012) included in the TorrentSuit 3.6 (Thermo Fisher Scientific/Life Technologies) software with default settings. Following best practice (GATK forum) guidelines, for each raw binary alignment file, duplicated reads were detected and removed using the software Picard v.1.69 tool MarkDuplicates (http://picard.sourceforge.net) (Van der Auwera *et al.* 2013). We further realigned reads in regions of potential insertions or deletions (indels) with the GATK tool (McKenna *et al.* 2010) Indel Realignment, and performed Base Quality Recalibration with the covariates read group, quality score, context size (3 bp) and cycle. Publically available genetic variation (SNPs and indels) from the Ensembl variation database (CanFam3.1, dog release 75) was used as “true positives” in base quality score recalibration, and variant calling Ensembl Variation Release 77 (Canis lupus familiaris) (ftp://ftp.ensembl.org/pub/release-77/variation/vcf/canis_familiaris/).

SNVs and indels were called simultaneously on the entire family trio using SAMtools (Li *et al.* 2009) and the GATK (McKenna *et al.* 2010) tool UnifiedGenotyper. Raw variant calls were filtered with VariantFiltration tool, using the standard hard filtering parameters (Van der Auwera *et al.* 2013): with mapping quality (MQ) < 40 and quality by depth (QD) < 2.0 for both SNVs and indels, (Read Position Rank Sum test) ReadPosRankSum < –8.0 for SNVs, and ReadPosRankSum < –20.0 for indels, Fisher strand (FS) > 60.0 for SNVs, and FS > 200.0 for indels.

### Detection of candidate mutations

The SNVs and indels were classified using gene predictions from Ensembl build version 75 with ANNOVAR (Wang *et al.* 2010). A custom Perl script was used to extract variants with the conditional filtering using the following criteria: autosomal recessive pattern of inheritance *i.e.*, parents heterozygous and the affected offspring homozygous for the variant allele. We used publicly available genetic variation in dogs (Axelsson *et al.* 2013) Ensembl Variation Release 77 (*Canis lupus* familiaris) (ftp://ftp.ensembl.org/pub/release-77/variation/vcf/canis_familiaris/) and our custom SNV data set derived from genome sequences of other dog breeds to extract novel/unknown candidates (or eliminate already known variants). To evaluate identified missense and nonsense mutations, SIFT (Sim *et al.* 2012) was used. The coverage at the identified positions were then inspected manually using IGV (Robinson *et al.* 2011; Thorvaldsdóttir *et al.* 2013).

### Sanger sequencing

Amplification primers for seven missense mutations predicted as deleterious by SIFT were designed using the online version (http://biotools.umassmed.edu/bioapps/primer3_www.cgi) of Primer3 (Rozen and Skaletsky 2000). The primers were constructed with M13 forward or reverse tails (Supporting Information, Table S2). Amplification and sequencing was done using the BigDye Direct Cycle Sequencing Kit (Thermo Fisher Scientific/Life Technologies) on a ProFlex PCR System (Applied Biosystems) and GeneAmp PCR System 9700 (Applied Biosystems). Four nanograms of genomic DNA were used in a 10 µl amplification reaction with an annealing temperature of 60° in accordance with the manufacturers’ protocol. Cycle sequencing was performed with both the forward (5′-TGTAAAACGACGGCCAGT-3′) and reverse (5′-CAGGAAACAGCTATGACC-3′) sequencing primers on an ABI 3500XL DNA Analyzer (Applied Biosystems). The sequences were then analyzed using CodonCode Aligner v5.0.2 (CodonCode Corporation).

### Conservation in mammals

For each of the candidate mutations, multiple sequence alignments at DNA and protein level were performed. For DNA level we extracted human alignments for 100 vertebrates available at the Santa Cruz Genome Browser (UCSC) for each of the corresponding dog positions. For protein alignments, we first extracted the protein sequences from the HomoloGene NCBI site for each of the gene containing the candidate substitution. Next, we used the standalone version of MUSCLE (Edgar 2004) software using the default parameters for multiple sequence alignments.

### Data availability

The data sets (three BAM files and one VCF file) supporting the results of this article are available in the European Nucleotide Archive (ENA) repository, [study accession number: PRJEB12301, http://www.ebi.ac.uk/ena/data/view/PRJEB12301.

## Results

### Next generation sequencing

Sequence libraries were constructed from genomic DNA prepared from a family trio consisting of two healthy parents and their HFH-affected offspring. The sequence reactions from each library were loaded on two Ion PI chips, yielding an average of 158 million reads per dog, of which 98% were aligned to the reference genome (CanFam3.1). The average read length was 138 bp, and, on average, 21.8 Gb sequence was generated for each dog (Table S1). Alignment of the reads to the canine reference genome resulted in a genomic coverage of 9.2X per individual dog, with around 96% of the genome and 91% of the exome covered with at least one read.

In total, 3,726,772 SNVs and 2,474,309 indels were detected using GATK v.2.7 Unified Genotyper tool (Boycott *et al.* 2013), of which 3,449,902 SNVs and 198,165 indels remained after standard hard filtering.

### Functional annotation of sequence variants

**Figure 1:**
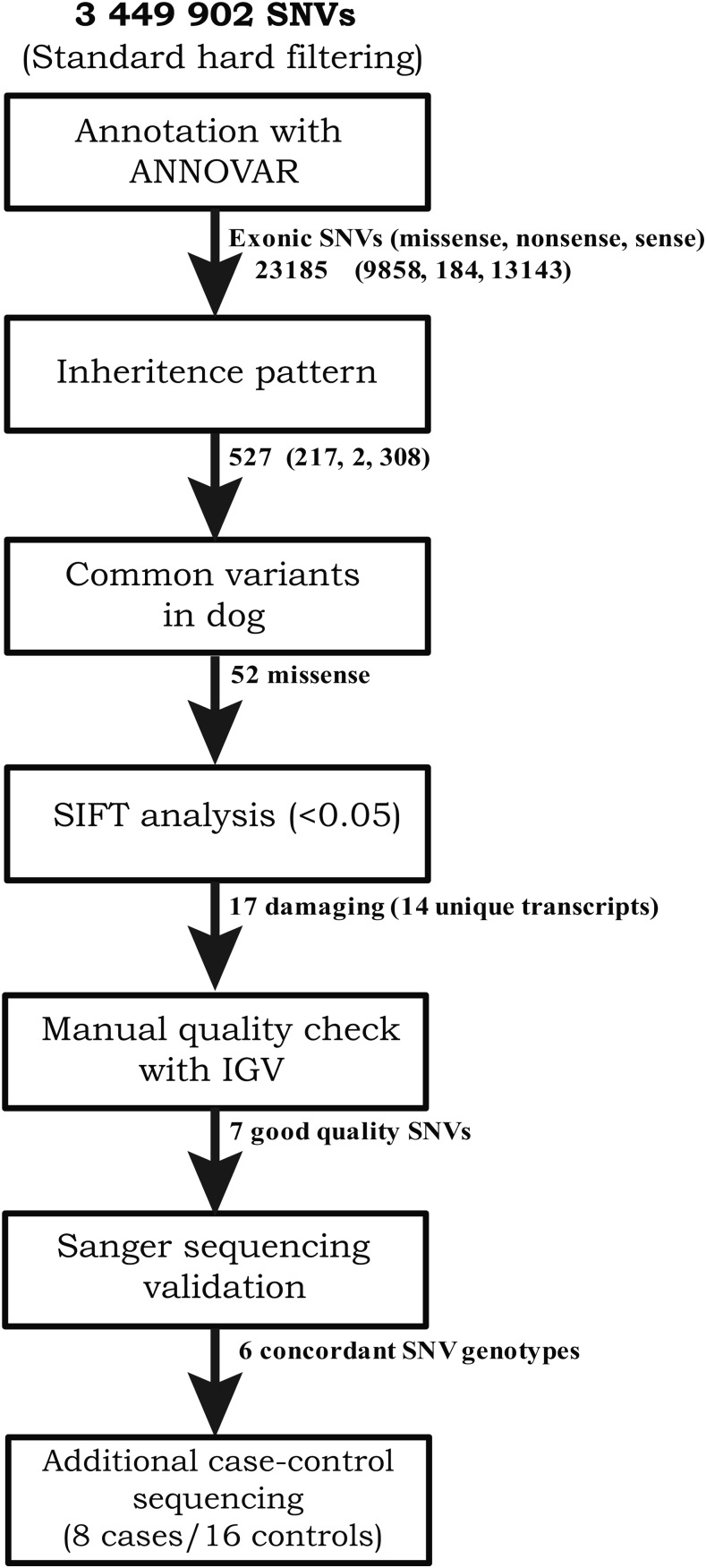
Schematic representation of the filtering pipeline used to evaluate genetic variants.

Functional annotation of the detected sequence variants was performed. In total, 23,185 SNVs were found to be exonic, and, of those substitutions, 9858 were missense, 184 were nonsense, and 13,143 were silent (Figure 1); 491 indels were located in exons. Next, a conditional filtering was applied based on a pattern of inheritance where both parents were heterozygous for a substitution and the affected offspring was homozygous. This resulted in the detection of 527 SNVs that fulfilled this conditional inheritance pattern. Of those, 217 were found to be missense, two nonsense, and 308 were silent substitutions. We identified 15 indels that followed the conditional inheritance pattern, and all but one was predicted to result in a frameshift of the open reading frame. A total of 165 missense substitutions, both nonsense substitutions, and all 14 indels were found to be known common variants, thus we were left with 52 missense substitutions for functional prediction analysis using SIFT (Sim *et al.* 2012).

The results of SIFT analysis suggested that 17 of the 52 missense substitutions were deleterious (SIFT score < 0.05). Single missense substitutions were found in 11 genes, and three genes had two missense substitutions, resulting in a total of 14 candidate genes to be further evaluated for their potential association with HFH. Using IGV and manual inspection, nine of the 17 missense substitutions were eliminated due to genotype errors or insufficient coverage. The remaining eight nonsynonymous substitutions were located in the following functional genes: *TDRD6*, *USP4*, *TACC2*, *DLGAP2*, *GRAPL* (two substitutions), *FAM83G*, and *PDILT*. These were further considered as candidate causative coding variants associated with development of HFH.

### Validation of candidate causative coding variants

**Figure 2:**
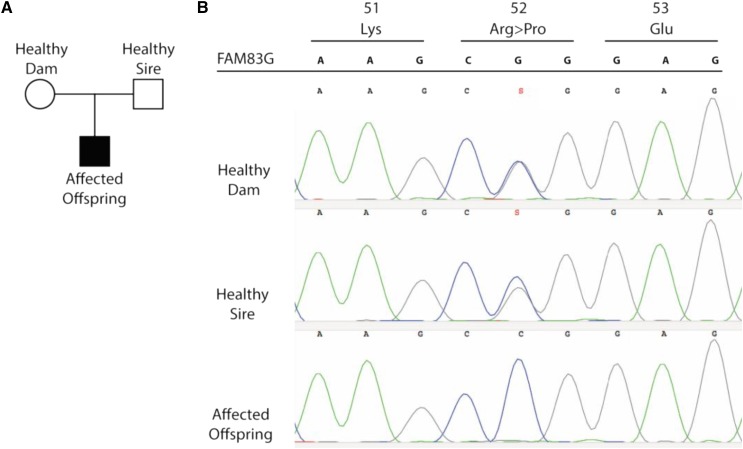
The family trio used for WGS and the validation of the missense variant at *FAM83G* (c155G > C, p.R52P) by Sanger sequencing. (A) The genome of Kromfohrländer dogs from a family trio consisting of an offspring affected by HFH and both its healthy parents was sequenced. (B) Both parents were heterozygous G/C (S) at position 155 of the *FAM83G* gene, and the affected offspring was homozygous C/C, resulting in a change at amino acid position 52 from an arginine to proline. The nucleotide sequence of codons 51–53 from the canine genome reference sequence is shown at the top.

To validate the NGS data from the family trio, the same three individuals were genotyped using Sanger sequencing of the eight candidate causative coding variants (Figure 2A and Table 1). All variants except the missense substitution present in the *TACC2* gene were confirmed to be heterozygous in the parents, and homozygous in the affected offspring, thus leaving six candidate genes with potential causative coding variants to be further investigated.

**Table 1:** Validation of the genotypes obtained by WGS of the family trio

Gene	*GRAPL*	*GRAPL*	*FAM83G*	*PDILT*	*DLGAP2*	*TDRD6*	*USP4*
Position	chr5: 41014230	chr5: 41014231	chr5: 41055619	chr6: 24951743	chr37: 30832376	chr12: 14853811	chr20: 39951727
Affected offspring	T/T	T/T	C/C	T/T	T/T	T/T	T/T
Healthy sire	G/T	C/T	C/G	G/T	C/T	C/T	G/T
Healthy dam	G/T	C/T	C/G	G/T	C/T	C/T	G/T

Genotyping was made by Sanger sequencing of the seven candidate causative variants located in six different genes: *GRAPL*, *FAM83G*, *PDILT*, *DLGAP2*, *TDRD6*, and *USP4*. The genes and their respective chromosomal positions are indicated above. Genotypes obtained from the affected offspring, the healthy sire, and the healthy dam are indicated.

To further explore the concordance of the predicted deleterious variants in *GRAPL*, *FAM83G*, *PDILT*, *DLGAP2*, *TDRD6*, and *USP4*, an additional eight HFH-affected and 16 healthy Kromfohrländer dogs were genotyped using Sanger sequencing. All dogs affected by HFH were found to be homozygous for the variants in the *GRAPL* and *FAM83G* genes. In contrast, the missense substitutions found in the other four genes were nonconcordant in affected and healthy dogs (Table 2), and could consequently be excluded as being causative for HFH.

**Table 2:** Genotyping by Sanger sequencing of the seven candidate causative variants located in six different genes to validate the concordance among cases

Differences in:	GRAPL	GRAPL	FAM83G	PDILT	DLGAP2	TDRD6	USP4
Position	chr5:41,014,230	chr5:41,014,231	chr5:41,055,619	chr6:24,951,743	chr37:30,832,376	chr12:14,853,811	chr20:39,951,727
Case 1	T/T	T/T	C/C	G/T	C/T	C/C	T/T
Case 2	T/T	T/T	C/C	G/T	C/C	C/C	G/G
Case 3	T/T	T/T	C/C	G/T	C/T	C/T	G/G
Case 4	T/T	T/T	C/C	T/T	C/C	T/T	G/G
Case 5	T/T	T/T	C/C	G/T	C/T	C/T	T/T
Case 6	T/T	T/T	C/C	G/G	C/T	T/T	T/T
Case 7	T/T	T/T	C/C	G/G	C/T	C/T	G/G
Case 8	T/T	T/T	C/C	G/G	C/T	C/T	G/T
Control 1	G/G	C/C	G/G	G/T	T/T	C/T	T/T
Control 2	G/G	C/C	G/G	G/G	T/T	C/T	G/G
Control 3	G/G	C/C	G/G	G/T	T/T	C/T	G/G
Control 4	G/G	C/C	G/G	G/G	C/T	C/C	G/G
Control 5	G/T	C/T	C/G	G/T	C/T	C/T	G/G
Control 6	G/T	C/T	G/G	G/T	C/T	C/T	G/T
Control 7	G/G	C/C	G/G	G/G	C/T	C/C	G/G
Control 8	G/G	C/C	G/G	G/G	C/T	C/C	G/G
Control 9	G/T	C/T	C/G	G/G	T/T	C/C	G/T
Control 10	G/G	C/C	G/G	G/G	C/T	C/C	G/T
Control 11	G/T	C/T	C/G	G/T	C/C	C/T	G/G
Control 12	G/G	C/C	G/G	G/T	C/T	C/T	G/G
Control 13	G/G	C/C	G/G	G/T	C/C	C/T	G/G
Control 14	G/G	C/C	G/G	G/G	C/T	C/C	G/G
Control 15	G/G	C/C	G/G	G/T	C/T	C/T	G/G
Control 16	G/G	C/C	G/G	G/T	C/C	C/T	G/T
CanFam3.1	G/G	C/C	G/G	G/G	C/C	C/C	G/G

The *GRAPL*, *FAM83G*, *PDILT*, *DLGAP2*, *TDRD6*, and *USP4* genes and their respective chromosomal positions with genotypes are indicated. The eight additional affected individuals (cases 1–8), the 16 healthy controls (controls 1–16), and in the CanFam3.1 reference genome sequence are indicated to the left.

#### Description of disease-associated variants:

The missense variant in the *FAM83G* gene (Ensembl gene ID: ENSCAFG00000018245), was a G to C transversion at position chr5:41,055,619 (CanFam3.1) that was predicted to cause a nonsynonymous substitution in the first exon of *FAM83G*. This missense variant (c.155G > C; p. R52P) substitutes a positively charged arginine to a neutral proline (Figure 2B). In a comparison with available genome sequence data from 29 placental mammals (Lindblad-Toh *et al.* 2011), it was found that the arginine residue at p.52 was completely conserved. A prediction analysis using HOPE (Venselaar *et al.* 2010) suggested that the substitution is damaging to the FAM83G protein.

The affected dogs were also homozygous for two substitutions adjacent to each other at positions chr5:41,014,230-41,014,231 in the *GRAPL* gene (Ensembl gene ID: ENSCAFG00000029170). However, based on comparison to other species, the Ensembl annotation of exon 1 of the *GRAPL* gene appears to be incorrect. Only in dogs, this part of the gene is annotated as being protein coding. Furthermore, we analyzed NGS sequence data from 49 additional dog genomes of which 48 were derived from dogs unaffected by HFH and one dog affected by the disease. The genotype distribution from this analysis revealed that nine dogs, one Kromfohrländer case and eight dogs from other breeds (Beagle, Entlebucher mountain dog, Eurasier, Siberian husky, Lagotto Romagnolo, and Sloughi), were homozygous for the mutant allele (TT/TT). In addition, nine dogs were heterozygous (GC/TT). The majority (31 dogs) of the other dogs were homozygous for the GC variant (data not shown).

## Discussion

GWAS have for almost a decade been the main strategy for identifying disease-associated alleles in canine Mendelian disorders. In the present proof-of-concept study, we show that WGS of a canine family trio can be used successfully to identify a candidate mutation for HFH, which is an autosomal recessive disease in Kromfohrländer dogs.

Today, the goal of “the $1000 genome” is close to being achieved (Hayden 2014), and NGS has already transformed human genetic research (Kilpinen and Barrett 2013). In studies of human monogenic diseases in small pedigrees, candidate mutations have been identified with WGS or WES (*e.g.*, Glazov *et al.* 2011; Harakalova *et al.* 2012; Ng *et al.* 2009, 2010; Roach *et al.* 2010). A striking example of a family trio-based WES approach was the identification of a nonsynonymous mutation in the *ABCC9* gene that is causative of the Cantú syndrome—a dominant disorder characterized by cardiac defects, congenital hypertrichosis (abnormal hairiness), and distinctive facial features. The missense mutation was detected by exome sequencing of one affected child and both his unaffected parents (Harakalova *et al.* 2012). Similarly, applying WGS on a family quartet where both offspring were affected by two autosomal recessive disorders (Miller syndrome and primary ciliary dyskinesia) and the parents were unaffected resulted in the identification of four candidate genes for these two Mendelian disorders (Roach *et al.* 2010).

HFH, also known as digital hyperkeratosis (DH), is an orthokeratotic palmoplantar hyperkeratosis with autosomal recessive inheritance, first described clinically in Irish Terriers (Binder *et al.* 2000), and also affecting the Kromfohrländer breed. Here we show that a missense variant in *FAM83G* (c155G > C, p.R52P) is associated with HFH in Kromfohrländer dogs. The *FAM83G* gene encodes a protein with only limited knowledge concerning its function. However, FAM83G was recently identified as a novel SMAD1 interactor that was shown to be a modulator for bone morphogenetic protein (BMP)-dependent signaling (Vogt *et al.* 2014). The amino acid residue arginine (R) at position 52 is highly conserved among 29 placental mammals (Lindblad-Toh *et al.* 2011), and the substitution p.R52P in FAM83G is likely to be damaging. However, to conclusively define the biological effect of this nonsynonymous substitution, functional experiments are needed. The identification of *FAM83G* (c155G > C, p.R52P) as a likely candidate for HFH is in agreement with results published by Drögemüller and colleagues based on a GWAS of 42 Kromfohrländer dogs (13 cases and 29 controls), and 31 Irish Terriers (10 cases and 21 controls) followed by deep WGS of one affected dog (Drögemüller *et al.* 2014). In that study, a 611-kb haplotype was identified that was shared by all the affected dogs. WGS of one affected Kromfohrländer dog that was compared to 46 genomes from healthy dogs of different breeds revealed the causative allele at the *FAM83G* locus.

In the present study, we undertook an alternative approach to identify the genetic variant associated with HFH in a population of Swedish Kromfohrländer dogs. Without prior GWAS, and directly using WGS of a single family trio (an affected offspring and both its healthy parents), we confirmed that the *FAM83G*:c155G > C (p.R52P) variant is associated with HFH. To our knowledge, this is the first time WGS of a canine family trio has been used successfully to identify a disease-associated allele.

### Conclusions

We conclude that the missense variant *FAM83G*:c155G > C (p.R52P) is associated with HFH in Kromfohrländer dogs. Our result independently confirms a previous report using GWAS of a case-control population followed by WGS of a single affected dog, and show that WGS of a family trio with two healthy carrier parents and one affected offspring is an efficient strategy for the identification of rare alleles associated with canine Mendelian disorders. This approach circumvents the challenge of sampling sufficient number of cases and controls needed to achieve statistical power in GWAS.

## Supplementary Material

Supporting Information
