# Supplementary material for: Whole-Genome Sequencing of a Canine Family Trio Reveals a FAM83G Variant Associated with Hereditary Footpad Hyperkeratosis
Source: G3 (Bethesda). 2016 Jan 8;6(3):521–7. doi: 10.1534/g3.115.025643 (PMC4777115; doi:10.1534/g3.115.025643)
Supplement: Supporting Information [file supp_6_3_521__index.html]

Whole-Genome Sequencing of a Canine Family Trio Reveals a FAM83G Variant Associated with Hereditary Footpad Hyperkeratosis — Whole-Genome Sequencing of a Canine Family Trio Reveals a FAM83G Variant Associated with Hereditary Footpad Hyperkeratosis — Supporting Information 

# Whole-Genome Sequencing of a Canine Family Trio Reveals a *FAM83G* Variant Associated with Hereditary Footpad Hyperkeratosis

## Supporting Information for Sayyab *et al.*, 2016

**Files in this Data Supplement:**

- Table S1 - Summary alignment statistics. (.pdf, 69 KB)
- Table S2 - Primers used for Sanger sequencing. (.xls, 27 KB)
