# Supplementary material for: Whole-Genome Sequencing of a Canine Family Trio Reveals a FAM83G Variant Associated with Hereditary Footpad Hyperkeratosis
Source: G3 (Bethesda). 2016 Jan 8;6(3):521–7. doi: 10.1534/g3.115.025643 (PMC4777115; doi:10.1534/g3.115.025643)
Supplement: Supporting Information [file supp_g3.115.025643_TableS1.pdf]

Table S1. Summary Alignment statistics. Proportion of aligned reads and average depth for each chip and each individual is given.

	Average per chip	On average per individual
Total Number of Bases [Gbp]	10.9	21.8
Total Number of Reads	79006173	158012346
Mean Length [bp]	138	138
proportion of Aligned bases	97%	97%
proportion of mapped reads	98%	98%
Average base coverage depth (raw)	4.6	9.2
